# Injuries are not accidents: towards a culture of prevention

**Published:** 2014-09-30

**Authors:** Francisco Javier Bonilla-Escobar, María Isabel Gutiérrez

**Affiliations:** Instituto Cisalva, School of Public Health, Universidad del Valle, Cali, Colombia

**Keywords:** Injuries, wounds and injuries, accident prevention, traffic accidents

## Abstract

Injuries are the result of an acute exposure to exhort of energy or a consequence of a deficiency in a vital element that exceeds physiological thresholds resulting threatens life. They are classified as intentional or unintentional. Injuries are considered a global health issue because they cause more than 5 million deaths per year worldwide and they are an important contributor to the burden of disease, especially affecting people of low socioeconomic status in low- and middle-income countries. A common misconception exists where injuries are thought to be the same as accidents; however, accidents are largely used as chance events, without taken in consideration that all these are preventable. This review discusses injuries and accidents in the context of road traffic and emphasizes injuries as preventable events. An understanding of the essence of injuries enables the standardization of terminology in public use and facilitates the development of a culture of prevention among all of us.

## Abstract (es)

Las lesiones son el resultado de la exposición aguda a energía o consecuencia de la insuficiencia de un elemento vital que sobrepasan los umbrales fisiológicos e impiden la vida. Se clasifican en intencionales o no intencionales. Son consideradas un problema de salud pública a nivel mundial debido a que causan más de 5 millones de muertes por año en el mundo y por la carga de enfermedad que generan, afectando principalmente a población de bajos recursos en países de bajos y medianos ingresos. Un error común existe cuando las lesiones son consideradas como accidentes, sin embargo los accidentes están dados por el azar mientras que las lesiones son prevenibles. Esta es una revisión en torno a las lesiones y los accidentes para la comprensión de las lesiones en el tránsito como eventos prevenibles en pro de la estandarización de la terminología en salud pública y en seguridad vial para una cultura de prevención.

## Introduction

Injuries have traditionally been known as "accidents" or random and unavoidable events. In recent decades, the understanding of the factors that determine the nature of injuries has changed this concept and has rendered the term "accident" inaccurate. Injuries are instead described as preventable events with major consequences on public health and represent a significant global issue 1 ^,^ 2.

According to the World Health Organization (WHO), injuries may be self-inflicted or caused by road traffic events, disasters, interpersonal violence, drowning, fires, wars, poisonings, and falls 1. Injuries cause more than 5 million deaths per year worldwide and account for nearly 9% of global mortality and 16% of all disabilities. It is estimated that over 90% of deaths worldwide that result from injuries occur in low and middle income countries. Affecting primarily the young and economically active population, injuries cause incalculable costs for the health, legal, and social systems 1 ^,^ 2. 

In order to effectively manage and prevent injuries, it is important to identify the factors that can cause and influence them 2. To date, there have been several initiates with aims to prevent injuries, including the World Report on Violence and Health in 2002 and the World Report on Road Traffic Injury Prevention in 2004. As the leading causes of injury worldwide, violence results in more than 1.6 million deaths per year while road traffic events are responsible for 1.2 million deaths per year 1 ^,^ 3 ^,^ 4. Despite the various initiatives for reducing injuries, morbility and mortality rates do not reflect the desired outcomes. As a result, injuries remain an important cause of morbidity and mortality 5. 

The keystone for prevention is the identification and understanding of the problem; therefore, it should be understood that injuries have identifiable causes and are not simply the result of chance or "bad luck" 6. According to Baker *et al*., injuries are defined as the consequence of acute exposure to energy 7. This can be in the form of mechanical, thermal, electrical, chemical, or radiation energy and exists in amounts that exceed the threshold of physiological tolerance. In addition, the authors state that Injury may also result from a vital element deficiency (drowning, strangulation, freezing, etc.) 7.

The Oxford English Dictionary defines "accident" as an unfortunate incident that happens unexpectedly and unintentionally, typically resulting in damage or injury" 8. In Colombia, the National Traffic Code defines "accident" (*accidente*) as an "event that is typically involuntarily generated by at least one vehicle in movement, causing damage to people and property involved in it (...)" *("evento generalmente involuntario, generado al menos por un vehículo en movimiento, que causa daños a las personas y bienes involucrados en él (...)"*) 9. If injuries and road traffic events are not limited to actions determined by fate or luck and instead have causes that are predictable and preventable, it should be assumed that they are not accidental 10.

Injury research has shown that injuries can be analyzed and avoided; therefore, the word "accident" is not appropriate when it comes to describing preventable events. Thus, a review of the misuse of the term "accident" in its description of road traffic events is necessary to promote a culture of awareness and injury prevention.

There are multiple theories about accidents with the major ones originating from the field of engineering. These stem from the prevention of industrial accidents and most of them are explanatory and predictive 11. This approach attributes "accidents" mostly to the human factor where man intervenes, designs, and causes most of the artifacts and situations involved in an accident^12^. An example of this is road traffic events where, from an engineering perspective, there is a human impact on most of the factors involved (roads, vehicles and rescue measures). Most analyses of accidents conclude that the failure to follow protocol is the most frequent cause of accidents in these situations.

Road traffic events, defined by collisions involving moving vehicles, deserve a re-evaluation of the terminology used to describe them since this influences discussions and approaches to health and public safety. An event that is preventable, understandable, and even predictable cannot be called an event of chance 4.

## Evolution of injury prevention

Measures to control injuries have varied and have arisen from rigorous, diverse, and time consuming studies. The first person to discuss injuries as a problem of medical ecology was Gordon in 1949. He published the first analysis of injuries from an epidemiological perspective and described injuries as being epidemic with seasonal variations, time trends, and different geographic and socioeconomic distributions resembling factors of infectious diseases 13 ^,^ 14.

Given these first steps, it is understandable that the distribution of injuries was not random in time, place, and person, and therefore nor in its causes 14. Subsequently, the discussion of causal agents based on the concepts of infection began and understanding these became the main target for prevention. Gordon's contributions in 1949 did not adequately cover these causal agents so, in the same year, King suggested that injuries became more frequent with increases in various types of stressors. These stressors were later identified as specific factors necessary for injuries and included non-specific factors such as aging, disease and nutritional deficiencies 15.

Since 1942, due to the work of De Haven, injury control was opened to the modern age, focusing on the importance of the damage caused by the exchange of energy and collision conditions (not only the speed but also the impact) as determinants of injury 16 ^,^ 17. In 1961, Gibson, an experimental psychologist, was the first to clearly define the specific agents of injuries, attributing the cause of the injuries to the transmission and exchange of mechanical, thermal, radiant, chemical or electrical energy 18.

With the emphasis of injury in causes, Leavell and Clark developed in 1954 the concepts of primary, secondary and tertiary prevention to emphasize the various prevention strategies that can be adopted for a health event. Primary prevention includes methods to prevent the occurrence whereas secondary prevention includes methods for early detection and routing. Tertiary prevention includes methods to reduce the negative health impact of the event 19.

Haddon Jr., a physician and engineer, continued the discussion of injury with the interpretation of the energy vector and the susceptibility to this transmission. In an analogy to the epidemiological model, there is a person susceptible to injury (host) who interacts and is affected by some form of energy (an agent specific and necessary). The structure in which the adverse event develops (environment) determines the amount of transferred energy and subsequent injury characteristics and severity 14.

In the sixties, Robertson and Baker introduced the active and passive prevention terms to distinguish an individual's participation in preventing the development of disease. These concepts encouraged the expansion of public health measures to control diseases and injuries 14 ^,^ 20 ^,^ 21. In the case of traffic related injuries, this approach resulted in the manufacturing of automobiles that provided occupant protection with increased individual liability when a road traffic injury occurred 22. This was also reflected in changes in road infrastructure which have separated the different types of road users (pedestrians, cyclists, motorcyclists and public transport system) to reduce conflicts between users and avoid complicated decision making during travel.

Subsequently, Haddon developed a network for injury analysis based on the host, the environment, and the physical and social context in which the injury occurs. This is widely known as the Haddon matrix where these aspects are evaluated over time in phases spanning the pre-event, event, and post-event 14 ^,^ 23. This approach allowed the analysis of injury prevention levels, including changes in behavior, environment, and public policy^24^^,^ 25. Currently, it is a tool extrapolated to different areas of knowledge, particularly in health, and it is useful in analyzing situations for public policies formulation 24.

With the above conceptualization, various organizations have rallied around the definitions of injury in health. The Center for Disease Control and Prevention in the United States (CDC), heeding the call of public health, recognizes that injuries are not accidents; they are predictable and exhibit repetitive patterns. This has subsequently initiated processes of epidemiological demonstration and injury control to determine effective interventions to prevent injury 26 ^,^ 27.

In the second half of the twentieth century, "accident research" drew strength from evidence such as that provided by Haddon. Authors like Svanström initiated the Safe Communities movement to make injuries evident as events caused by factors such as environment, human, organization, and society. With the aim of positioning the subject of injuries on the public agenda and to take preventive actions to reduce its impact, WHO and CDC promote the premise that "there are no accidents" 28. 

The concept of Safe Community was developed in Sweden and initially implemented in Falköpin (1975) and later in Lidköping (1984) to recognize preventive actions that were carried out by the community. These included, in particular, actions to reduce deaths and injuries in children under 14 years of age; actions were accompanied by monitoring systems to identify the effect of interventions 29. The Falköpin program reduced the incidence of injuries in 4 years (1978-1981) by 34%; in Lidköping there were registered annual reductions in the incidence of injuries by 2.4% and 2.1% in boys and girls, respectively. With these studies, prevention strategies and the need to implement surveillance systems for identifying and managing injuries are included in the public agenda. This allows focusing on the different forms of injury and promotes safer communities in terms of road safety and violence prevention, among others 30.

## Changing the microchip: injuries are not accidents 

Houk, in 1986, wrote that implementing preventative practices would reduce injuries and costs associated with traffic events by 75% and reduce injuries at home by 50%. These figures have not been reached; thus, the prevention of injuries does not appear to be among the highest priorities of public health and this may be in part explained by the conception of injuries as accidents 26 ^,^ 31.

An example of the influence of words is evident in the term "recreational drugs". These substances have caused thousands of deaths and many related injuries in road traffic events and through violence; therefore, the term can be considered an oxymoron because it speaks of something harmful to take to recreate. In a similar sense, when referring to injuries, the incorrect use of the terms "accidents" and "traffic accidents" also represents an oxymoron as these terms describe random events that are supposed to happen in a planned environment and that can be preventable 31.

Another approach that supports the disuse of the word "accident" can be taken from the perspective of "accidents" in terms of "natural disasters" for which it has been shown that the effect of the development of society and human behavior is one of its main triggers 32. Thus, in this example, the word "accident" or "natural disaster" would be incorrectly used given its predictable etiology and thus the definition of the phenomenon, its study, and its approach would be affected.

Awareness and dissemination of the concept "injuries are not accidents" has taken years to reach the public health community. It is ironic that the word "accident" in articles referring to preventable events is still published. It is noteworthy that in the Medical Subject Headings (MeSH Terms) the word "accident" is still included as part of public health terminology, including the terms "accident prevention, safety, home accidents, occupational accidents, and traffic accidents".

The evidence that injuries are preventable is overwhelming but the impact of current injury prevention approaches could be greater if the use of the word "accident" is discontinued and instead replaced by the terms "injury" or "event". This would allow, among other things, an awareness of these events and quantification of their impact on the general population when injuries are assumed to be preventable and not part of a random scenario. 

The scientific community is a key part of this process of change in the use of the term "accident". Authors of research articles and the editorial boards of scientific journals may propose changes in terminology and encourage discussion. For example, the *British Medical Journal *in 2001 banned the term "accident" in their publications 33. Nevertheless, the clearest example is certainly the road safety policy "Vision Zero", adopted by the Swedish Parliament, with which it was generated a reduction in traffic deaths and injuries than any other intervention had achieved 34. With actions like this, the public health community will understand that injuries are preventable and, perhaps with all the researchers, community and stake holders support we can develop a culture of prevention. By refraining from considering preventable events as "accidents", only then can we accomplish the objective of reducing the incidence of injuries and fatalities in traffic events.
